# Supplementary material for: Coronavirus-Specific Antibody and T Cell Responses Developed after Sputnik V Vaccination in Patients with Chronic Lymphocytic Leukemia
Source: Int J Mol Sci. 2022 Dec 27;24(1):416. doi: 10.3390/ijms24010416 (PMC9820366; doi:10.3390/ijms24010416)
Supplement: Supplementary file 1 [file ijms-24-00416-s001.zip › Supplementary Figures.pdf]

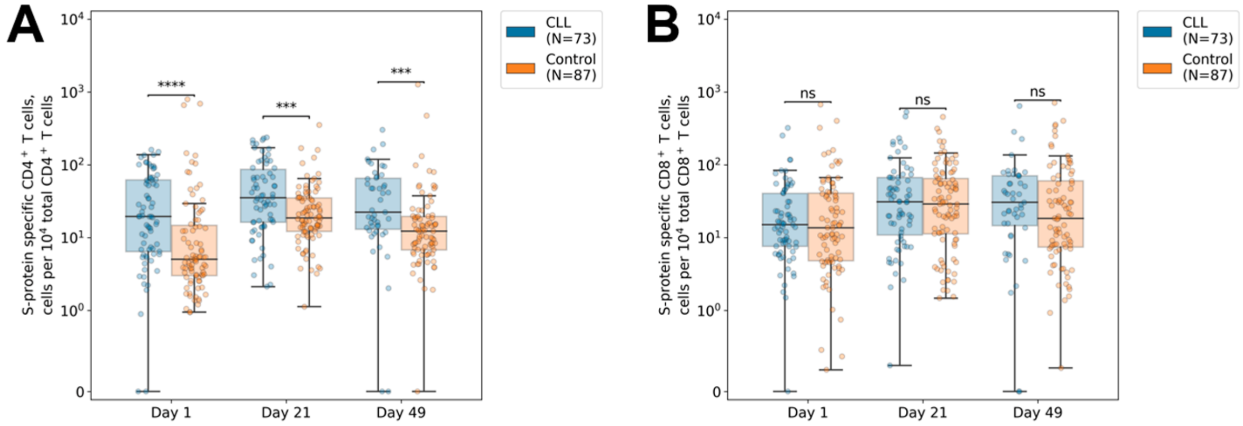

Supplementary Figure S1. Dynamics of the SARS-CoV-2-specific CD4+ and CD8+ T cell response. Immune response was evaluated among CD4+ (A) and CD8+ (B) T lymphocytes using flow cytometry prior to the vaccination on day 1 (d1), prior to the administration of the second component on day 21 (d21), and 28 days after second component administration (d49). A symmetric logarithm (symlog) scale was used for the y-axis, with the range from 0 to the first axis tick being in linear scale, and the rest of the range in logarithmic scale. p-values > 5.00e-02 are marked with ‘ns’; 1.00e-02 < p-values ≤ 5.00e-02 are marked with ‘*’; 1.00e-03 < p-values ≤ 1.00e-02 with ‘**’; 1.00e-04 < p-values ≤ 1.00e-03 with ‘***’; and p-values ≤ 1.00e-04 with ‘****’ (two-sided Mann–Whitney U test).

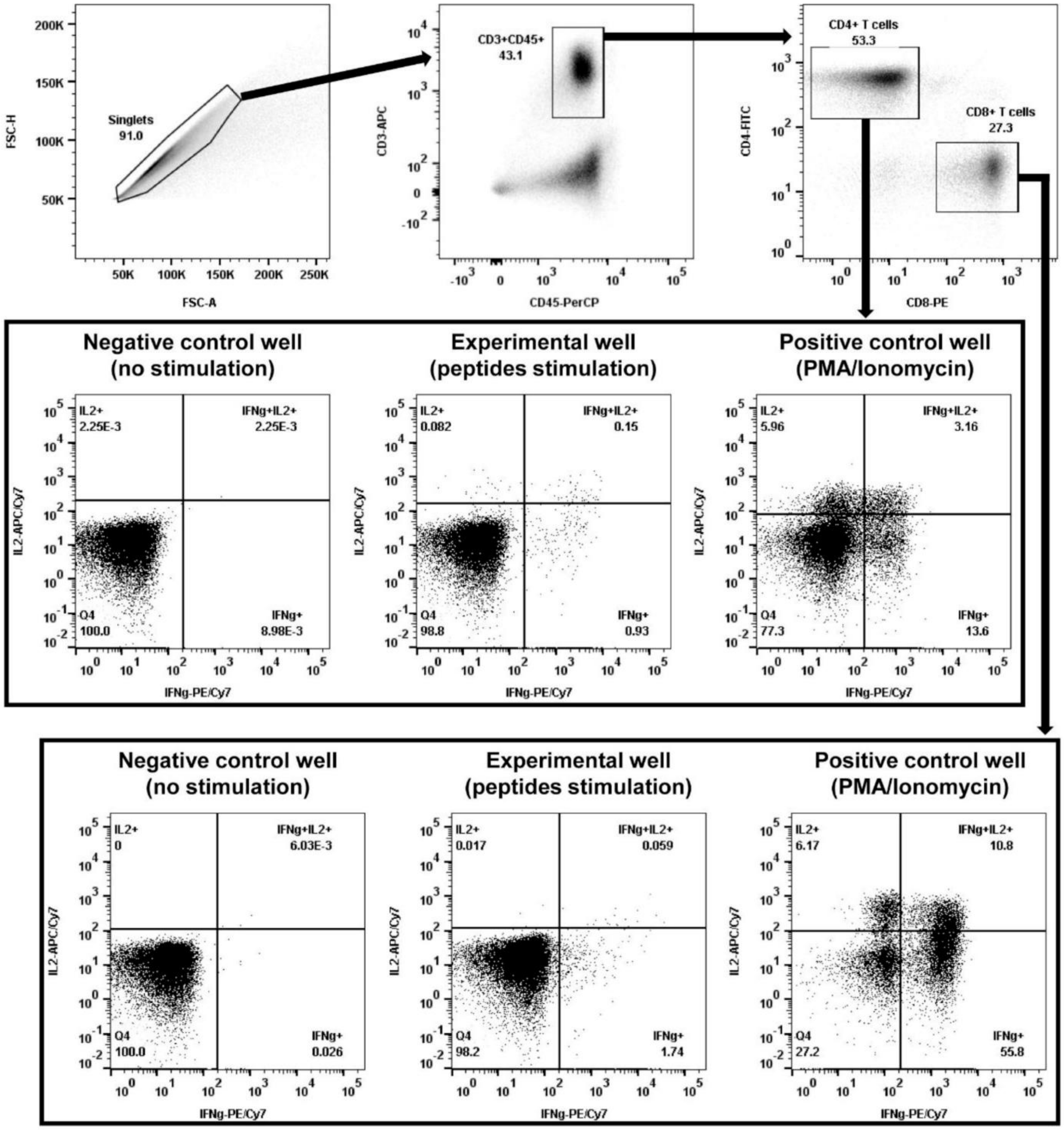

Supplementary Figure S2. Gating strategy used for flow cytometry analysis. Cells were sequentially gated on singlets (top left panel), CD3+CD45+ cells (top middle panel), CD4+ and CD8+ T-lymphocytes (top right panel). The expression of IFNγ and IL2 was analyzed in CD4+ (middle panel) and CD8+ (bottom panel) T cells. For each donor three wells were analyzed: unstimulated (negative) control, cells stimulated with SARS-CoV-2 peptides, and cells stimulated with PMA/Ionomycin (positive control). Representative dot plots from a single healthy donor are shown.
