# Supplementary material for: Coronavirus-Specific Antibody and T Cell Responses Developed after Sputnik V Vaccination in Patients with Chronic Lymphocytic Leukemia
Source: Int J Mol Sci. 2022 Dec 27;24(1):416. doi: 10.3390/ijms24010416 (PMC9820366; doi:10.3390/ijms24010416)
Supplement: Supplementary file 1 [file ijms-24-00416-s001.zip › Supplementary Tables.pdf]

Supplementary Table S1. Dynamics of the antibody response estimated among CLL patients.

Grouping parameter		Day 1				Day 21				Day 49			
		N	median [IQR]	pval	qval	N	median [IQR]	pval	qval	N	median [IQR]	pval	qval
**iBTK-containing treatment**	**YES**	47	0.7 [0.2-12.9]	0.19	0.32	47	2.9 [0.2-119.7]	0.46	0.48	36	5.7 [1.0-495.6]	0.46	0.4
	**NO**	24	0.6 [0.1-20.5]			24	2.8 [0.3-152.0]			16	10.8 [0.8-429.7]		
**Combination therapy with anti-CD20**	**YES**	14	0.4 [0.2-2.7]	0.22	0.33	14	0.4 [0.2-2.5]	**0.03**	0.17	9	1.0 [0.6-2.6]	**0.034**	0.23
	**NO**	57	1.0 [0.2-18.1]			57	6.7 [0.3-240.8]			43	12.1 [1.1-1155.0]		
**Venetoclax-containing treatment**	**YES**	23	0.7 [0.2-10.8]	0.46	0.48	23	0.5 [0.2-187.8]	0.22	0.26	15	2.5 [0.7-11.5]	0.11	0.4
	**NO**	48	1.0 [0.1-22.7]			48	4.4 [0.4-119.4]			37	16.3 [1.0-1494.2]		
**Total IgG > 5 g/L**	**YES**	49	1.0 [0.2-21.6]	0.15	0.3	49	7.2 [0.4-240.8]	**0.019**	0.17	37	16.4 [1.2-1494.2]	**0.023**	0.18
	**NO**	20	0.3 [0.2-2.6]			20	0.5 [0.2-10.8]			15	1.6 [0.4-11.0]		
**Total IgM > 0.41 g/L**	**YES**	29	0.4 [0.1-32.6]	0.12	0.26	29	6.7 [0.4-137.8]	0.24	0.17	21	44.7 [2.5-388.9]	**0.038**	0.26
	**NO**	40	1.1 [0.2-10.4]			40	1.5 [0.2-120.7]			31	2.6 [0.4-1094.3]		
**Total IgA > 0.8 g/L**	**YES**	34	1.4 [0.2-29.9]	0.11	0.26	34	37.2 [0.5-833.2]	**0.012**	0.17	25	61.2 [1.2-3592.9]	**0.028**	0.17
	**NO**	35	0.4 [0.2-2.8]			35	1.0 [0.2-15.8]			27	2.6 [0.6-22.3]		
**Age > 70 years**	**YES**	19	1.2 [0.2-21.0]	0.4	0.46	19	1.8 [0.3-222.6]	0.45	0.17	16	1.0 [0.5-20.3]	**0.025**	0.26
	**NO**	52	0.7 [0.2-14.9]			52	3.3 [0.2-119.4]			36	16.3 [1.6-1510.1]		
**Unmutated IGHV genes**	**YES**	53	0.4 [0.1-3.3]	**0.03**	0.17	53	1.0 [0.2-38.4]	0.19	0.49	39	3.6 [0.8-68.0]	0.48	0.49
	**NO**	10	2.1 [0.6-100.5]			10	15.0 [0.7-263.0]			7	6.6 [0.6-1087.2]		
**17p deletion**	**YES**	23	0.7 [0.2-12.9]	0.3	0.4	23	8.4 [0.3-239.3]	0.14	0.33	18	5.7 [1.3-2898.6]	0.22	0.21
	**NO**	45	0.7 [0.2-13.8]			45	1.2 [0.2-38.4]			31	3.6 [0.8-42.6]		
**Male sex**	**YES**	40	0.8 [0.2-4.7]	0.37	0.45	40	1.1 [0.3-92.5]	0.33	0.4	24	5.3 [0.6-183.8]	0.28	0.25
	**NO**	31	0.7 [0.1-23.9]			31	3.7 [0.2-130.1]			28	9.7 [1.1-985.4]		
**Number of previous therapy lines > 2**	**YES**	17	0.4 [0.1-2.0]	0.11	0.26	17	0.9 [0.2-7.2]	0.17	0.32	14	2.3 [0.9-20.5]	0.18	0.46
	**NO**	54	1.0 [0.2-31.0]			54	4.4 [0.3-212.8]			38	14.2 [1.0-1324.6]		

S-protein specific IgG titers were evaluated using the automated ARCHITECT i1000SR analyzer with compatible reagent kit (Abbott, USA) and presented as BAU/mL. N, number of patients per group; IQR, interquartile range; pval – p-level estimated using two-sided Mann–Whitney U test; qval – false discovery rate q-values estimated using the Benjamin–Hochberg (BH) procedure. Values below a threshold of 0.05 are marked in bold.

Supplementary Table S2. Dynamics of the T cell response among CLL patients estimated using ELISpot.

Grouping parameter		Day 1				Day 21				Day 49			
		N	median [IQR]	pval	qval	N	median [IQR]	pval	qval	N	median [IQR]	pval	qval
**iBTK-containing treatment**	**YES**	47	286.7 [18.3-976.7]	**0.00019**	**0.0084**	47	910.0 [303.3-1348.3]	**0.0011**	**0.017**	34	930.0 [462.5-1443.3]	**0.002**	0.1
	**NO**	24	16.7 [3.3-88.3]			24	80.0 [17.5-595.8]			16	96.7 [42.5-194.2]		
**Combination therapy with anti-CD20**	**YES**	14	45.0 [0.8-150.8]	**0.032**	0.12	14	260.0 [31.7-722.5]	**0.033**	0.42	8	251.7 [66.7-1452.5]	0.26	0.44
	**NO**	57	170.0 [13.3-823.3]			57	643.3 [110.0-1420.0]			42	658.3 [115.8-1377.5]		
**Venetoclax-containing treatment**	**YES**	23	170.0 [15.0-1273.3]	0.12	0.31	23	690.0 [218.3-1153.3]	0.49	0.44	15	596.7 [205.0-1443.3]	0.33	0.44
	**NO**	48	95.0 [10.0-463.3]			48	403.3 [80.0-1329.2]			35	633.3 [90.0-1361.7]		
**Total IgG > 5 g/L**	**YES**	49	143.3 [10.0-860.0]	0.41	0.46	49	620.0 [76.7-1230.0]	0.5	0.37	36	683.3 [105.8-1450.0]	0.18	0.21
	**NO**	20	105.0 [15.8-423.3]			20	520.0 [142.5-1395.0]			14	498.3 [65.8-1110.0]		
**Total IgM > 0.41 g/L**	**YES**	29	96.7 [10.0-846.7]	0.34	0.44	29	550.0 [80.0-1213.3]	0.22	0.5	22	658.3 [108.3-1325.8]	0.49	0.37
	**NO**	40	153.3 [15.8-740.0]			40	713.3 [106.7-1396.7]			28	596.7 [86.7-1443.3]		
**Total IgA > 0.8 g/L**	**YES**	34	110.0 [4.2-810.8]	0.21	0.37	34	608.3 [80.0-1193.3]	0.21	0.46	25	683.3 [103.3-1313.3]	0.4	0.44
	**NO**	35	170.0 [16.7-746.7]			35	550.0 [131.7-1480.0]			25	596.7 [106.7-1470.0]		
**Age > 70 years**	**YES**	19	13.3 [5.0-105.0]	**0.0023**	**0.017**	19	80.0 [45.0-670.0]	**0.0041**	**0.011**	15	90.0 [38.3-596.7]	**0.00069**	**0.011**
	**NO**	52	203.3 [16.7-955.0]			52	781.7 [179.2-1431.7]			35	1020.0 [223.3-1481.7]		
**Unmutated IGHV genes**	**YES**	53	136.7 [10.0-823.3]	0.21	0.37	53	620.0 [96.7-1230.0]	0.45	0.3	38	708.3 [185.0-1443.3]	0.11	0.44
	**NO**	10	55.0 [7.5-181.7]			10	306.7 [153.3-1171.7]			7	90.0 [68.3-676.7]		
**17p deletion**	**YES**	23	170.0 [73.3-940.0]	**0.029**	0.12	23	946.7 [303.3-1368.3]	0.068	0.1	16	1196.7 [212.5-1572.5]	**0.021**	0.3
	**NO**	45	20.0 [10.0-406.7]			45	326.7 [76.7-1213.3]			31	400.0 [81.7-981.7]		
**Male sex**	**YES**	40	108.3 [10.0-485.8]	0.27	0.42	40	543.3 [102.5-1195.0]	0.38	0.49	24	615.0 [105.8-1433.3]	0.46	0.45
	**NO**	31	136.7 [10.0-1005.0]			31	620.0 [88.3-1368.3]			26	673.3 [95.8-1377.5]		
**Number of previous therapy lines > 2**	**YES**	17	170.0 [16.7-523.3]	0.35	0.44	17	430.0 [70.0-1153.3]	0.21	0.32	13	226.7 [30.0-1123.3]	0.13	0.32
	**NO**	54	95.0 [10.0-753.3]			54	666.7 [110.0-1367.5]			37	733.3 [106.7-1430.0]		

IFNγ-expressing T cells were evaluated using the ELISpot assay (see Materials and Methods section in main text) and presented as standard spot forming units (SFU) per 106 PBMC. N, number of patients per group; IQR, interquartile range; pval – p-level estimated using two-sided Mann–Whitney U test; qval – false discovery rate q-values estimated using the Benjamin–Hochberg (BH) procedure. Values below a threshold of 0.05 are marked in bold.

Supplementary Table S3. Dynamics of the T cell response among CLL patients estimated using flow cytometry.

Grouping parameter		Day 1				Day 21				Day 49			
		N	median [IQR]	pval	qval	N	median [IQR]	pval	qval	N	median [IQR]	pval	qval
**iBTK-containing treatment**	**YES**	48	30 [8-69]	**0.0048**	0.21	46	48 [26-91]	**0.017**	0.32	32	41 [14-71]	**0.066**	0.45
	**NO**	25	11 [3-23]			24	17 [9-48]			15	18 [12-34]		
**Combination therapy with anti-CD20**	**YES**	15	20 [5-27]	0.12	0.32	14	30 [13-48]	0.13	0.32	7	16 [12-33]	0.1	0.45
	**NO**	58	19 [7-67]			56	39 [17-91]			40	28 [14-70]		
**Venetoclax-containing treatment**	**YES**	23	20 [5-81]	0.37	0.45	23	39 [16-76]	0.46	0.45	15	33 [15-73]	0.34	0.29
	**NO**	50	19 [7-62]			47	35 [16-84]			32	20 [12-65]		
**Total IgG > 5 g/L**	**YES**	50	20 [7-67]	0.13	0.32	47	48 [17-98]	**0.045**	0.35	32	32 [13-74]	0.16	0.45
	**NO**	20	14 [6-39]			20	25 [14-42]			15	18 [13-47]		
**Total IgM > 0.41 g/L**	**YES**	30	20 [6-59]	0.41	0.45	28	29 [13-67]	0.14	0.45	21	22 [14-66]	0.37	0.32
	**NO**	40	19 [7-67]			39	39 [19-108]			26	22 [13-62]		
**Total IgA > 0.8 g/L**	**YES**	34	20 [6-66]	0.34	0.45	32	52 [14-94]	0.28	0.45	23	21 [12-61]	0.24	0.29
	**NO**	36	17 [7-53]			35	33 [16-59]			24	28 [16-66]		
**Age > 70 years**	**YES**	21	15 [6-34]	0.1	0.32	21	28 [14-51]	0.074	0.3	15	19 [9-44]	**0.047**	0.3
	**NO**	52	20 [7-66]			49	42 [16-104]			32	37 [15-70]		
**Unmutated IGHV genes**	**YES**	55	15 [5-64]	0.3	0.45	52	32 [14-68]	0.1	0.49	35	22 [12-70]	0.49	0.45
	**NO**	10	21 [8-54]			10	113 [18-123]			7	16 [15-48]		
**17p deletion**	**YES**	23	15 [7-74]	0.4	0.45	22	40 [23-59]	0.48	0.32	15	19 [12-37]	0.14	0.45
	**NO**	47	19 [6-62]			46	32 [15-91]			29	41 [13-73]		
**Male sex**	**YES**	42	19 [7-60]	0.38	0.45	42	38 [16-88]	0.4	0.45	22	40 [13-65]	0.27	0.4
	**NO**	31	20 [6-63]			28	32 [16-74]			25	19 [14-64]		
**Number of previous therapy lines > 2**	**YES**	17	19 [5-35]	0.13	0.32	16	36 [26-56]	0.41	0.44	12	18 [15-48]	0.22	0.49
	**NO**	56	20 [7-67]			54	34 [15-101]			35	24 [13-71]		

CD4+ T cells were evaluated using the flow cytometry assay (see Materials and Methods section in main text) and presented as coronavirus-specific cells per 104 total CD4+ T cells. N, number of patients per group; IQR, interquartile range; pval – p-level estimated using two-sided Mann–Whitney U test; qval – false discovery rate q-values estimated using the Benjamin–Hochberg (BH) procedure. Values below a threshold of 0.05 are marked in bold.
